# Triptolide protects against podocyte injury in diabetic nephropathy by activating the Nrf2/HO-1 pathway and inhibiting the NLRP3 inflammasome pathway

**DOI:** 10.1080/0886022X.2023.2165103

**Published:** 2023-03-20

**Authors:** Chenlei Lv, Tianyang Cheng, Bingbing Zhang, Ke Sun, Keda Lu

**Affiliations:** a Department of Nephrology, The First Affiliated Hospital, Zhejiang Chinese Medical University, Hangzhou, Zhejiang, China; b College of Pharmaceutical Sciences, Zhejiang Chinese Medical University, Hangzhou, Zhejiang, China; c Department of Nephrology, The Third Affiliated Hospital, Zhejiang Chinese Medical University, Hangzhou, Zhejiang, China

**Keywords:** Diabetic nephropathy, MPC5, triptolide, NLRP3 inflammasome, Nrf2, oxidative stress

## Abstract

**Objectives:** Diabetic nephropathy (DN) is the most common microvascular complication of diabetes mellitus. This study investigated the mechanism of triptolide (TP) in podocyte injury in DN.

**Methods:** DN mouse models were established by feeding with a high-fat diet and injecting with streptozocin and MPC5 podocyte injury models were induced by high-glucose (HG), followed by TP treatment. Fasting blood glucose and renal function indicators, such as 24 h urine albumin (UAlb), serum creatinine (SCr), blood urea nitrogen (BUN), and kidney/body weight ratio of mice were examined. H&E and TUNEL staining were performed for evaluating pathological changes and apoptosis in renal tissue. The podocyte markers, reactive oxygen species (ROS), oxidative stress (OS), serum inflammatory cytokines, nuclear factor-erythroid 2-related factor 2 (Nrf2) pathway-related proteins, and pyroptosis were detected by Western blotting and corresponding kits. MPC5 cell viability and pyroptosis were evaluated by MTT and Hoechst 33342/PI double-fluorescence staining. Nrf2 inhibitor ML385 was used to verify the regulation of TP on Nrf2.

**Results:** TP improved renal function and histopathological injury of DN mice, alleviated podocytes injury, reduced OS and ROS by activating the Nrf2/heme oxygenase-1 (HO-1) pathway, and weakened pyroptosis by inhibiting the nod-like receptor (NLR) family pyrin domain containing 3 (NLRP3) inflammasome pathway. *In vitro* experiments further verified the inhibition of TP on OS and pyroptosis by mediating the Nrf2/HO-1 and NLRP3 inflammasome pathways. Inhibition of Nrf2 reversed the protective effect of TP on MPC5 cells.

**Conclusions:** Overall, TP alleviated podocyte injury in DN by inhibiting OS and pyroptosis *via* Nrf2/ROS/NLRP3 axis.

## Introduction

Diabetes mellitus (DM) is a chronic metabolic disorder chiefly characterized by hyperglycemia, and the incidence of DM is increasing worldwide [1]. Diabetic nephropathy (DN), the most common microvascular complication of DM, is primarily featured by albuminuria and progressive loss of kidney function [2,3]. Intrinsically, podocyte injury and loss are the main causes of proteinuria, and podocyte injury is mainly attributed to oxidative stress (OS) and inflammatory injury induced by high-glucose (HG) [4,5]. To date, the treatment and management strategies of DN mainly focus on reducing body weight, blood glucose, and blood pressure, and the commonly used first-line treatment is renin-angiotensin system inhibitors, including angiotensin-converting enzyme inhibitors or angiotensin receptor blockers [6,7]. Dapagliflozin (DAPA) is an inhibitor of sodium-glucose cotransporter-2 and is often used as the first choice for DN treatment in clinical practice [8,9]. However, these therapies have certain limitations and side effects. It is interesting to note that Chinese medicine has numerous targets and good potential in the treatment of DN [10]. Consequently, it is of great significance to find potent targets and therapeutic modalities for DN.

Recent evidence suggests that the production and accumulation of reactive oxygen species (ROS) caused by hyperglycemia and hyperlipidemia lead to OS, which is critical in DN [11,12]. In particular, excessive ROS can cause kidney damage by promoting lipid oxidation, thus resulting in podocyte injury and inflammation [13,14]. Nuclear factor-erythroid 2-related factor 2 (Nrf2), a key factor in cell regulation of OS, is the most sensitive signal for scavenging intracellular ROS to resist OS and is also one of the therapeutic targets of DN [15,16]. Interestingly, Nrf2 can inhibit the activation of Nod-like receptor (NLR) family pyrin domain containing 3 (NLRP3) inflammasome by scavenging ROS [17]. Inflammasomes are multi-protein complexes expressed in myeloid cells, present in the cytoplasm of many cell types, and can induce innate immune responses by sensing damage signals and microbial attacks [18]. NLRP3 inflammasome is the most widely studied complex, consisting of NLRP3, apoptosis-associated speck-like protein (ASC), and pro-caspase-1 [19]. Compelling evidence suggests the involvement of the NLRP3 inflammasome pathway in the pathological process of type 2 DM [20]. Meanwhile, the NLRP3 inflammasome pathway-mediated pyroptosis is essential in renal injury [21]. Recent research has been reported that Festine reduces DN-provoked podocyte injury by inhibiting NLRP3 inflammasome [22]. In particular, ROS is an important factor in NLRP3 inflammasome activation, and inhibition of ROS levels in cells can impede NLRP3 inflammasome activation [21,23]. The aforementioned studies imply that the Nrf2/ROS/NLRP3 pathway may be a therapeutic target for DN.

Triptolide (TP) is an alkaloid extracted from traditional Chinese medicine *Tripterygium wilfordii*, which has anti-inflammatory, antioxidant, hypoglycemic, lipid-lowering, and antitumor effects [24]. Cumulative evidence suggests the protective effect of TP on DN [25,26]. For instance, Han F et al. identified in their work that TP could inhibit the PDK1/Akt/mTOR pathway in human renal mesangial cells to protect against DN [27]. Hence, TP may be one of the candidate drugs for treating DN [28]. Considering all of this evidence, we hypothesized that TP can protect against DN podocyte injury by activating the Nrf2/HO-1 pathway to reduce ROS levels, inhibit the NLRP3 inflammatory pathway, and regulate OS and pyroptosis. The specific objective of this study was to investigate the therapeutic effect and mechanism of TP on DN, aiming to provide new insights into the pathogenesis and treatment of DN.

## Materials and methods

### Ethics statement

Animal experiments were approved by the Animal Ethics Committee of The Third Affiliated Hospital, Zhejiang Chinese Medical University (Approval number: IACUC-20210406-15), and adequate measures were taken to minimize the mouse number and pain or discomfort. The study was carried out under ARRIVE guidelines.

### Establishment of animal models

The male C57BL/6J mice aged 6–8 weeks were provided by Beijing Experimental Animal Research Center (Beijing, China) [Animal License No. SYXK (Beijing) 2021-0045]. The mice were fed with a standard diet and free drinking water and reared at 23 ± 1 °C under standard light/dark cycles and 60% humidity for 1 week. As stated previously [29], the DN mouse model was established by feeding with a high-fat diet (HFD) for 2 months and then intraperitoneally injecting with streptozotocin (STZ, 50 mg/kg, Solarbio, Beijing, China) for continuous 7 days. Normal mice were administered with an equal amount of sodium citrate buffer. Seven days after injection, fasting blood glucose (FBG) levels were measured using a glucose meter (Roche, Germany) and urine samples within 24 h were collected in a metabolic cage, followed by the determination of 24 h urine albumin (24 h UAlb) using the Bradford method. Mice with FBG ≥ 16.9 mmol/L and increased 24 h UAlb were considered DN mice.

### Group treatment of animals

Normal mice or successfully-established DN mice were assigned as follows, with 8 mice per group: (1) normal control (NC) group; (2) DN group; (3) DN + dimethyl sulfoxide (DMSO) group: DN mice were administrated with 1 mL normal saline containing 0.4% DMSO; (4) DN + TP group: DN mice were intragastrically administrated with 100 μg/kg/d TP (Yuanye Bio-Technology, Shanghai, China). The dosage of TP was determined based on previous research [30] and pre-experiments. The purity of TP determined by high-performance liquid chromatography purity was >98%. TP was dissolved in DMSO and diluted with normal saline; (5) DN + DAPA group: DN mice were intragastrically administrated with 1 mg/kg/d DAPA (Yuanye Bio-Technology) [31]. After 12 weeks of continuous treatment, all urine samples were collected in the metabolic cage for 24 h, and FBG and 24 h UAlb were determined the next morning. The survival rate of mice was shown in Table 1, and eventually, six surviving mice in each group were selected for subsequent analyses.

**Table 1.** Survival status of mice after 12 weeks of treatment.

Group	NC	DN	DN + DMSO	DN + TP	DN + DAPA
Number of survivals	8/8	6/8	7/8	8/8	8/8
Survival rate	100%	75%	87.5%	100%	100%

### Determination of serum indexes

Following previous work [32], serum indexes were determined. After treatment, the mouse weight was weighed and recorded. Mice were deeply anesthetized with 1% pentobarbital sodium (50 mg/kg body weight) and sacrificed by drawing cardiac blood. The blood samples were centrifuged at 1000 g for 5 min at 4 °C and the serum was collected. Part of the serum was used to detect serum creatinine (SCr) and blood urea nitrogen (BUN) in mice using an automated biochemical analyzer (ADVIA 1650 Chemistry System; Bayer, Leverkusen, Germany), and the rest of the serum was used for subsequent detection.

### Hematoxylin-eosin (H&E) staining

As previously described [33], after the mice were sacrificed, bilateral kidneys were removed, the left kidneys were weighed, and the ratio of kidney/body weight (K/B Weight) was calculated. Thereafter, the kidney was fixed with 10% paraformaldehyde in a refrigerator at 4 °C for 24 h, embedded in paraffin, and sliced into sections of 5 μm thickness. Sections were stained using H&E staining kits (G1120-100, Solarbio). Paraffined sections were regularly dewaxed and hydrated, stained with hematoxylin for 5 min, and then rinsed with tap water. Following differentiation with 1% hydrochloric acid ethanol for 5 s, the sections were rinsed with running water again and treated with 1% ammonia for 3–5 s. After washing with tap water, the sections were restained with eosin for 3 min, rinsed with water, dehydrated with gradient ethanol, cleared with xylene, and then sealed with neutral gum. The sections were observed and photographed under a light microscope (Olympus, Tokyo, Japan) and the pathological changes and the area of inflammatory infiltration in mouse kidney tissues were evaluated by Image Pro-Plus (Media Cybernetics, MD, USA). Five slices from each of the six mice in each group were collected, and each slice was randomly selected for 5 fields, with the results averaged.

### Terminal deoxynucleotidyl transferase-mediated dUTP-biotin nick end labeling (TUNEL) staining

The apoptosis in mouse kidney tissues was measured using TUNEL detection kits (C1098, Beyotime, Shanghai, China) [33]. Paraffined sections of mouse kidney tissue were routinely dewaxed, hydrated, washed with phosphate-buffered solution (PBS), and incubated with 20 μg/mL protease K at room temperature for 30 min. After soaking with PBS, sections were incubated with a mixture of enzyme solution and TUNEL standard solution (diluted at 1:10) at room temperature for 60 min. Next, sections were restained with hematoxylin, dehydrated with gradient ethanol, cleared with xylene for 2 min, and sealed with neutral gum. The sections were observed and photographed under a light microscope, and five fields were randomly selected from each section. Image Pro-Plus software was utilized to count the percentage of apoptotic cells (%), and the average value was calculated.

### Transmission electron microscope (TEM) observation

Following previous work [33,34], the 1 mm^3^ of mouse kidney tissue was fixed with 3.75% glutaraldehyde and 1% osmium acid. Subsequently, the tissues were embedded in epoxy resin and stained with uranyl acetate and citric acid. Afterward, TEM (Olympus, Tokyo, Japan) was used to observe the ultrastructural changes in mouse podocytes. Image-Pro Plus software was used to analyze the changes in the average number of podocytes and podocyte foot processes in glomerulus. Five slices from each of the six mice in each group were collected, and each slice was randomly selected for five fields, with the results averaged.

### Immunohistochemistry

As mentioned in prior research [35], the sections of mouse kidney tissues were incubated with 3% hydrogen peroxide to eliminate endogenous peroxidase activity and then heated using a microwave oven for antigen repair. Later, the sections were blocked with 5% bovine serum albumin at 37 °C for 30 min and incubated with diluted primary anti-Nephrin (1:2000, ab216341, Abcam, UK) overnight at 4 °C. After washing with PBS, the sections were incubated for 30 min with biotin-labeled immunoglobulin G (IgG) H&L secondary antibody (1:1000, ab207995) at 37 °C. Following washing with PBS, the sections were incubated for 15 min with horseradish peroxidase (HRP)-labeled streptomycin at room temperature, dripped with 3,3′-diaminobenzidine chromogenic solution (P0202, Beyotime) in dark conditions, and rinsed with tap water. The sections were restained with hematoxylin, dehydrated with gradient ethanol, cleared with xylene, and sealed with neutral gum, followed by observation and photography under a light microscope. Five visual fields were randomly selected for each section. Image Pro-Plus software was adopted to analyze the percentage of positive cells (%). The brown-yellow area was regarded as the positive expression, and the results were averaged.

### Cell culture

Conditional immortalized mouse podocytes MPC5 (Cell Bank of Chinese Academy of Sciences, Shanghai, China) were cultured in RPMI 1640 medium supplemented with 10% fetal bovine serum and 1% penicillin-streptomycin (Sigma-Aldrich, MI, USA) [32] and were induced with recombinant interferon-γ (Sigma-Aldrich) at 33 °C for proliferation. After 10–12 days, cells were cultured in a condition free of interferon-γ at 37 °C until the cells were fully differentiated into mature podocytes. After differentiation, cells were cultured in a constant temperature incubator with 5% CO_2_ at 37 °C.

### Cell grouping

As previously described [36], MPC5 cells at the logarithmic growth stage were harvested and grouped as follows: (1) blank group: treated with 5 mM glucose; (2) HG group: treated with 25 mM glucose; (3) HG + DMSO group: treated with 25 mM glucose and 0.1% DMSO; (4) HG + TP group: treated with 25 mM glucose and 10 μM TP (the administration method and dosage of TP were referred to the literature [37]); (5) HG + DAPA group: treated with 25 mM glucose and 2 μM DAPA, with the DAPA dosage referred to the literature [31]; (6) HG + DMSO + ML385: treated with 25 mM glucose, 0.1% DMSO, and 5 μM ML385 (Nrf2 inhibitor, ab287109, Abcam), with the concentration of ML385 referred to the instruction and reference [38]; (7) HG + TP + ML385: treated with 25 mM glucose, 10 μM TP, and 5 μM ML385; (8) HG + TP + si-NC: transfected with si-NC plasmid (GenePharma, Shanghai, China) for 24 h and then treated with 25 mM glucose and 10 μM TP; (9) HG + TP + si-Nrf2: transfected with si-Nrf2 plasmid (GenePharma) for 24 h and next treated with 25 mM glucose and 10 μM TP. After 48 h of treatment, MPC5 cells were collected for subsequent experimentation.

### 3-(4,5-Dimethylthiazol-2-yl)-2,5-diphenyltetrazolium bromide (MTT) assay

The cell viability was detected by MTT assay [39]. MPC5 cells were cultured in 96-well plates (1 × 10^4^ cells/well) and incubated with MTT solution (M405849-1Set, Aladdin, Shanghai, China) at a final concentration of 1 mg/mL for 4 h at 37 °C. Formazan crystal was dissolved with 150 μL/well of DMSO and the absorbance at 570 nm was measured with a microplate reader.

### Cell pyroptosis detection

The cell pyroptosis was examined by Hoechst 33342/propidium iodide (PI) double fluorescence staining kits (CA1120, Solarbio) [40]. After different treatments in groups, MPC5 cells at the logarithmic growth stage were cultured in 6-well plates and stained with 10 μL Hoechst 33342 solution at 37 °C for 10 min under dark conditions. Thereafter, the cells were stained with 5 μL PI in the dark at 25 °C for 15 min. Staining results were observed using a fluorescence microscope (Nikon 80i, Nikon, Japan) and images were collected.

### Immunofluorescent staining

Following previous work [41], cell slides were prepared and fixed with 4% paraformaldehyde. Following rinsing with PBS, the slides were sealed with goat serum at room temperature for 30 min and then incubated with the anti-Nephrin (1:500, ab216341, Abcam) overnight at 4 °C. The slides were washed in PBS with 0.05% Tween-20 three times and next incubated with goat anti-rabbit secondary antibody Alexa Fluor^®^ 594 IgG H&L (2 μg/mL, ab150080) in dark conditions. The nuclei were stained with 4′,6-diamidino-2-phenylindole and photographed under a fluorescence microscope.

### Measurement of ROS

ROS levels in kidney tissue or MPC5 cells were detected by 2′,7′-dichlorodihydrofluorescein diacetate (DCFH-DA) fluorescence probe and ROS levels in fresh frozen sections of mouse kidney tissue were measured using the kits (HR7814, Biolab, Beijing, China) [41]. In brief, the unfixed frozen kidney tissue sections of 10 μm thickness were added with 200 μL washing solution at room temperature, with the solution spreading over the surface of sections. After the sections were allowed to stand for 5 min, the washing solution was carefully aspirated and then 100 μL staining working solution was added dropwise. Following incubation for 30 min at 37 °C under conditions devoid of light, the staining solution was removed. Thereafter, sections were rinsed 2–3 times with PBS and mounted with glycerol, followed by the detection of fluorescence intensity under a fluorescence microscope. Additionally, ROS levels in MPC5 cells were measured using the kits (S0033; Beyotime). The collected cells were diluted with PBS, seeded into 6-well plates, and then incubated with DCFH-DA solution at a final concentration of 5 mM for 30 min at 37 °C. After washing with PBS, the fluorescence intensity of the samples was detected by a fluorescence microscope, with the emission wavelength set at 530 nm and the excitation wavelength set at 485 nm. The operation steps were carried out according to the kit instructions.

### Detection of OS-related indicators and inflammatory factors

Following previous work [34], kidney tissue homogenate or MPC5 cells were collected. Total protein was extracted using radioimmunoprecipitation assay lysate (W063-1-1, Jiancheng Bioengineering Institute, Nanjing, China). Protein concentration in the supernatant was measured using the bicinchoninic acid method (P0012S, Beyotime). Subsequently, the supernatant was diluted 20 times and 50 μL of diluted supernatant was collected to detect the content of the target protein. The levels of OS-related indexes malondialdehyde (MDA) (A003-1-2), superoxide dismutase (SOD) (A001-3-2), and glutathione (GSH) (A006-1-1) were detected by kits (Jiancheng). The mouse serum or MPC5 cells were collected, followed by determining the levels of inflammatory cytokines interleukin (IL)-1β (H002) and IL-18 (H015) by ELISA kits (Jiancheng).

### Western blotting (WB)

The total protein was extracted from mouse kidney tissues or cells using radioimmunoprecipitation assay lysate (W063-1-1, Jiancheng) [34]. After the protein concentration was measured using the bicinchoninic acid kits (P0012S, Beyotime), the proteins (40 μg) were isolated by 10% sodium dodecyl sulfate-polyacrylamide gel electrophoresis (SDS-PAGE) and transferred onto polyvinylidene fluoride membranes. Next, the membranes were blocked with 5% skim milk for 1 h and incubated with rabbit primary antibodies anti-Nephrin (1:1000, ab216341, Abcam), anti-Podocin (1:10000, ab181143), anti-Nrf2 (1:1000, ab92946), anti-heme oxygenase-1 (HO-1) (1:1000, ab68477), anti-NLRP3 (1:1000, ab270449), anti-ASC (1:5000, ab155970), anti-Pro Caspase-1 (1:200, ab238972), anti-Gasdermin D N-terminal domain (GSDMD-N) (1:1000, ab215203), and anti-glyceraldehyde-3-phosphate dehydrogenase (GAPDH) (1:1000, ab9485) overnight at 4 °C. After washing with PBS, the membranes were incubated with HRP-labeled goat anti-rabbit secondary antibody IgG H&L (1:20000, ab97051) at room temperature for 30 min. After that, the membranes were developed by enhanced chemiluminescence and then observed and photographed. Image-Pro Plus 6.0 (Media Cybernetics, Inc., MD, USA) was adopted to analyze the relative expression of different proteins, with GAPDH as an internal reference.

### Statistical analysis

SPSS 21.0 software (IBM Corp. Armonk, NY, USA) and GraphPad Prism 8.0 software (GraphPad Software Inc., CA, USA) were used for statistical analysis and plotting of data. Statistical data tested by Shapiro-Wilk were normally distributed and expressed as mean ± standard deviation (SD). The *t*-test was used for data comparison between two groups, one-way analysis of variance (ANOVA) was used for data comparison among multiple groups, and Tukey’s test was used for the *post-hoc* test. *p*-Value was obtained from the bilateral tests. *p* < 0.05 was considered statistically significant.

## Results

### TP improved renal function and histopathological injury in DN mice

**Figure 1.**
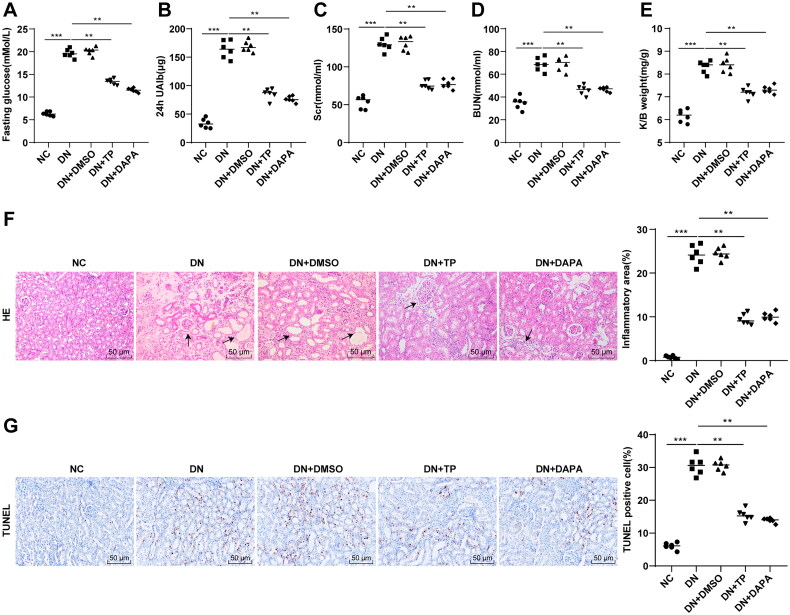
TP improved renal function and histopathological injury in DN mice. The DN mouse model was induced by HFD feeding combined with STZ injection. After 12 weeks of treatment with TP, (A) FBG was detected by glucose meter; (B) 24 h UAlb in mice was detected by Bradford method; (C,D) The serum contents of SCr and BUN in mice were detected using an automated biochemical analyzer; (E) The ratio of K/B weight was examined; (F) H&E staining was used to analyze the pathological changes of renal tissues in mice and to quantify the percentage of inflammatory areas; (G) TUNEL staining was used to detect the percentage of apoptosis in renal tissues. Data were expressed as mean ± *SD*, *N* = 6. One-way ANOVA was used for data comparison among multiple groups and Tukey’s test was used for the *post-hoc* test. *p*-Value was obtained from the bilateral tests. **p < 0.01, ***p < 0.001.

The DN mouse model was established by HFD combined with STZ injection, followed by treatment with TP, with DAPA as the positive control. FBG, 24 h UAlb, SCr, and BUN of mice were measured, and the K/B weight ratios of mice were calculated. Compared with the NC group, FBG, 24 h UAlb, Scr, and BUN in the DN group were increased (Figures 1(A–D), *p* < 0.001), as well as the ratio of K/B weight (Figure 1(E), *p* < 0.001). Furthermore, H&E staining manifested that the glomerular morphology of mice in the NC group was regular and the renal cell morphology was normal; however, the renal tissue cells in the DN group were swollen and morphologically altered, with increased inflammatory infiltration areas (Figure 1(F), *p* < 0.01). TUNEL staining showed that apoptosis of renal tissue cells was enhanced in the DN group (Figure 1(G), *p* < 0.001). Compared with the DN group, FBG, 24 h UAlb, SCr, and BUN in the DN + TP group were decreased (Figures 1(A–D), all *p* < 0.01), along with a decrease in K/B weight ratio to the normal range (Figure 1(E), *p* < 0.05). After TP treatment, the edema of mouse kidney tissue was alleviated, the cell morphology tended to be normal, the inflammatory infiltration area was reduced (Figure 1(F)), and the apoptosis in renal tissues was reduced in DN mice (Figure 1(G), *p* < 0.001). In parallel, DMSO conferred no obvious effect on DN mice and the therapeutic effect of TP was comparable to that of DAPA (Figures 1(A–G), all *p* > 0.05). Altogether, TP can improve renal function and histopathological damage in DN mice.

### TP alleviated podocyte injury in DN mice

**Figure 2.**
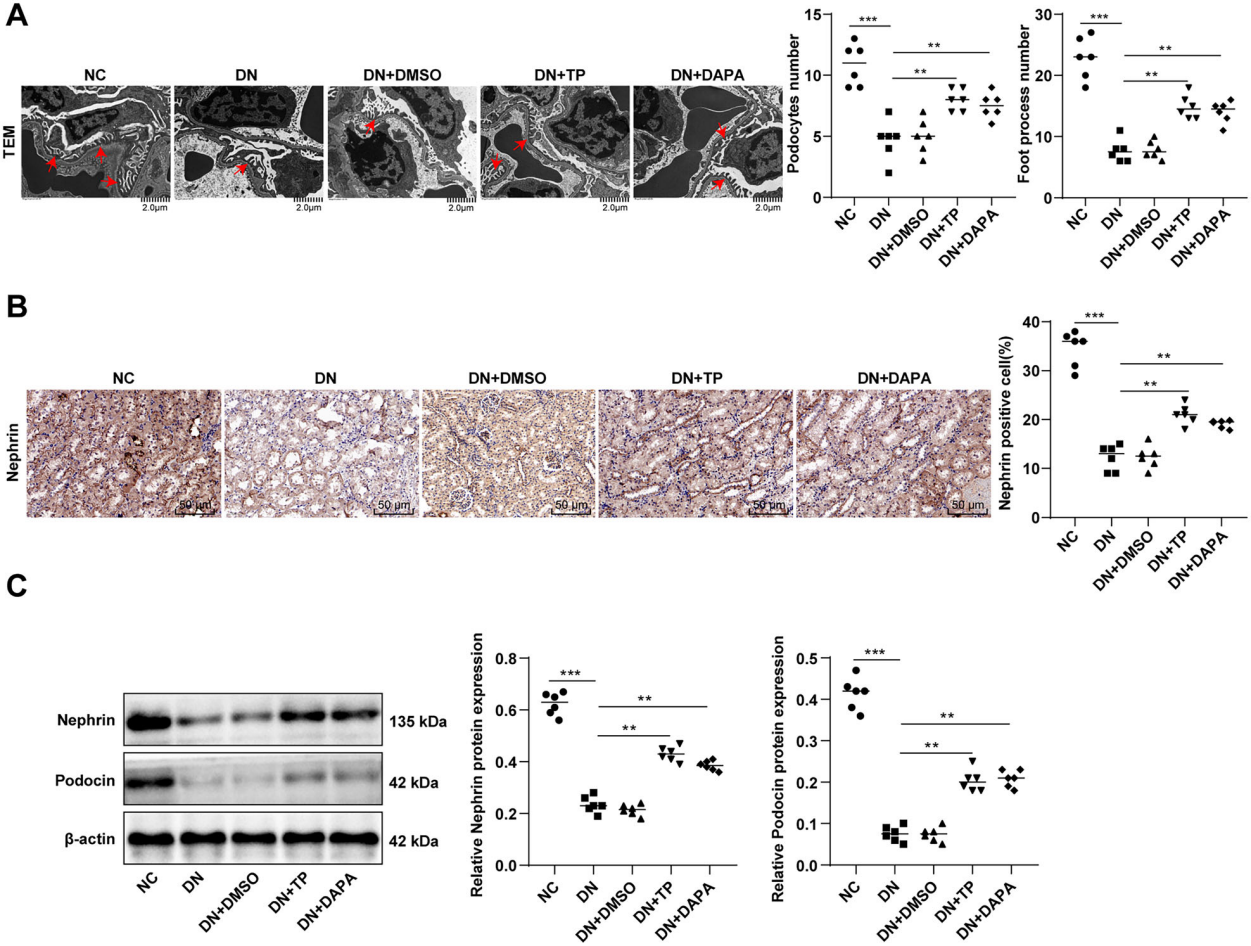
TP alleviated podocyte injury in DN mice. (A) The morphological and structural changes of podocytes in mouse kidney tissue were observed by TEM and the average number of podocytes and podocyte foot processes in glomeruli were quantitatively analyzed; (B) The number of Nephrin-positive cells was detected by immunohistochemistry; (C) The protein levels of Nephrin and Podocin in mouse kidney tissues were detected by WB. Data were expressed as mean ± *SD*, *N* = 6. One-way ANOVA was used for data comparison among multiple groups and Tukey’s test was used for the *post-hoc* test. **p < 0.01, ***p < 0.001.

The pathogenesis of DN and the production of proteinuria are extremely related to podocyte injury [42,43]. TEM observation revealed that the average number of podocytes and podocyte foot processes in glomerulus was decreased and the number of hiatus of foot processes was reduced in the DN group, with the disappearance of slit diaphragm (Figure 2(A)). Subsequently, immunohistochemistry discovered that DN mice had a reduced number of Nephrin-positive podocytes in kidney tissues (Figure 2(B), *p* < 0.001). WB manifested that the protein levels of Nephrin and Podocin in kidney tissues of DN mice were decreased (Figure 2(C), *p* < 0.001), indicating significant damage to renal podocytes in the DN group. After TP treatment, the number of podocytes, foot processes, and hiatus of foot processes was elevated, podocyte characteristics were partially restored (Figure 2(A)), Nephrin-positive cells were increased (Figure 2(B), *p* < 0.01), and the protein levels of Nephrin and Podocin were enhanced (Figure 2(C), both *p* < 0.05). Additionally, there was no significant impact of DMSO on podocytes and no significant difference between the DN + TP group and the DN + DAPA group (Figures 2(A–C), all *p* > 0.05). These results verified the protective effect of TP on glomerular podocytes.

### TP ameliorated OS injury and activated the Nrf2/HO-1 pathway in the renal tissue of DN mice

**Figure 3.**
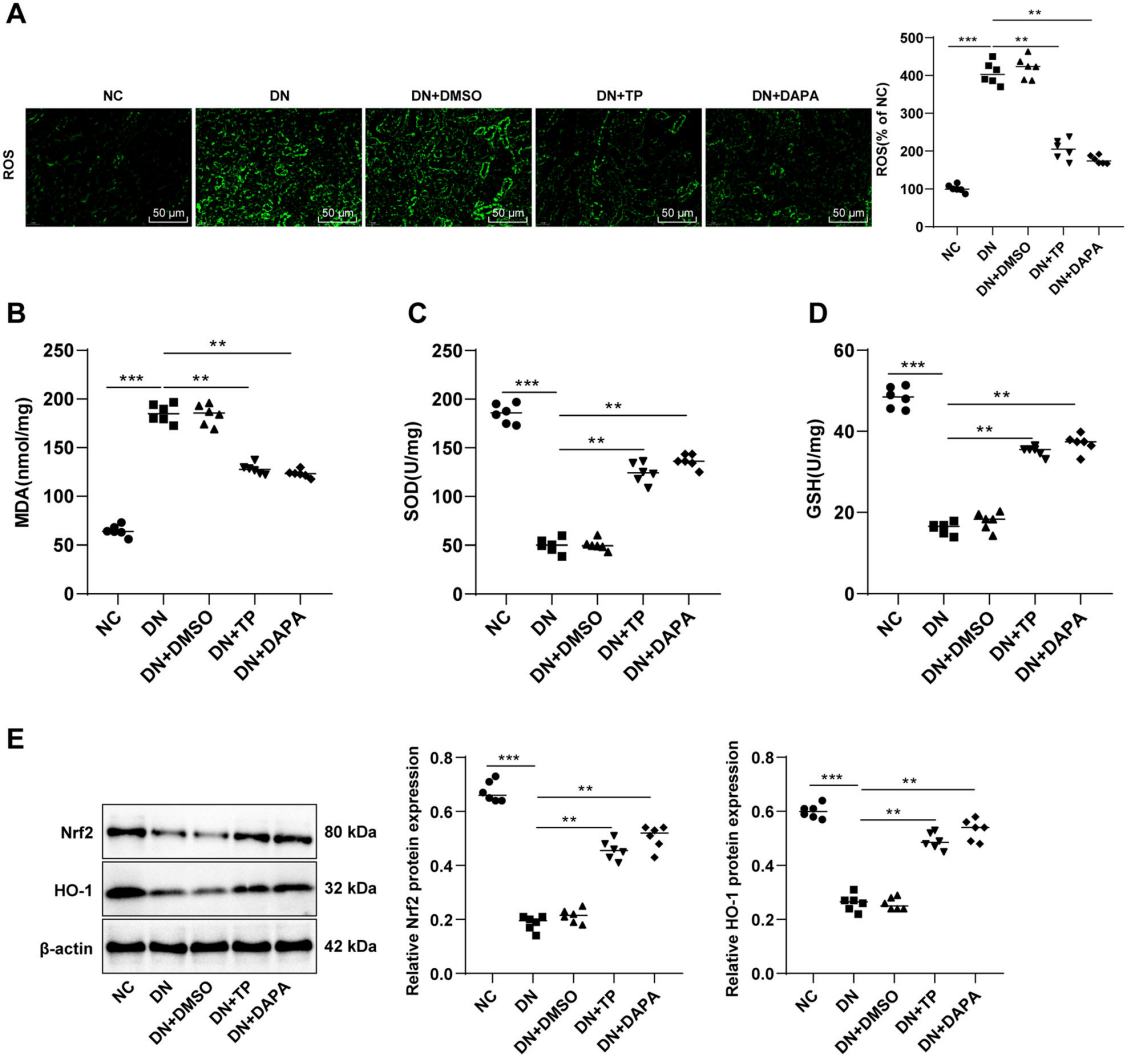
TP improved OS injury and activated the Nrf2/HO-1 pathway in renal tissue of DN mice. (A) DCFH-DA kits were used to detect ROS levels in mouse renal tissues; (B–D) The levels of OS-related enzymes, such as MDA, SOD, and GSH were detected; E: The levels of the Nrf2/HO-1 pathway-related proteins in mouse kidney tissues were detected by WB. Data were expressed as mean ± *SD*, *N* = 6. One-way ANOVA was used for data comparison among multiple groups and Tukey’s test was used for the *post-hoc* test. **p < 0.01, ***p < 0.001.

Evidence suggests the close association between DN occurrence and OS imbalance [11,12]. The ROS, MDA, SOD, and GSH levels in mouse renal tissue were determined. It was found that ROS and MDA levels were enhanced and SOD and GSH levels were decreased in the DN group (Figures 3(A–D), all *p* < 0.05). In comparison to the DN group, the DN + TP group had diminished ROS and MDA levels as well as increased SOD and GSH levels (Figures 3(A–D), all *p* < 0.05). These results uncovered that TP can ameliorate OS injury in the renal tissue of DN mice. It is noteworthy that the Nrf2/HO-1 pathway is the core pathway against OS [44,45]. WB assay unveiled that relative to the NC group, the protein levels of Nrf2 and HO-1 in the DN group were decreased, while TP activated the levels of Nrf2 and HO-1 (Figure 3(E), *p* < 0.05). In addition, DMSO exerted no significant effect on DN mice, and in comparison with the DN + DAPA group, DN + TP group showed no significant difference in the above indexes (Figures 3(A–E), all *p* > 0.05). In summary, TP may protect against DN by stimulating the Nrf2/HO-1 pathway to reduce the ROS level and alleviate OS injury.

### TP reduced pyroptosis of renal tissue in DN mice by inhibiting the NLRP3 inflammasome pathway

**Figure 4.**
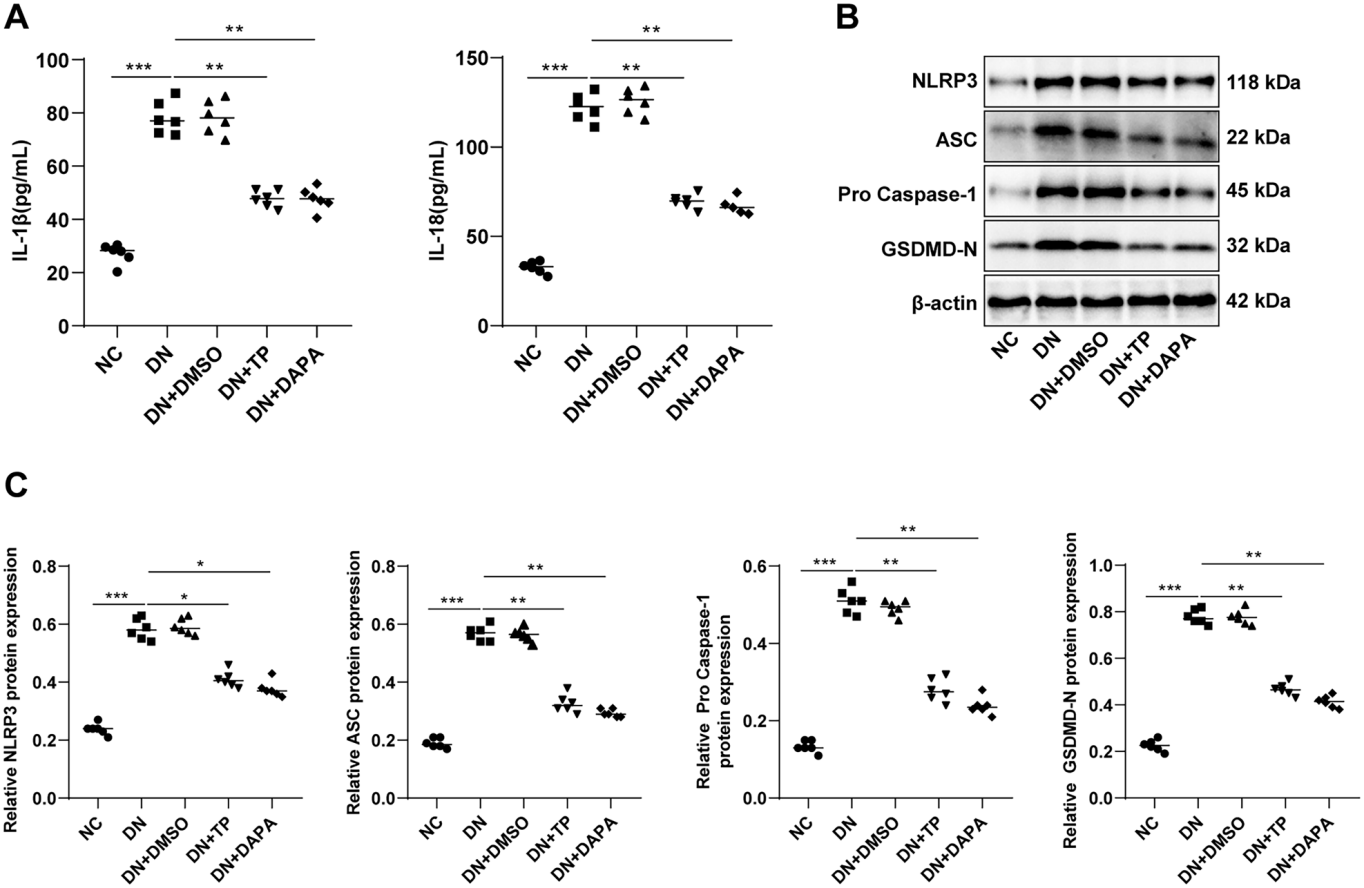
TP reduced pyroptosis of renal tissue in DN mice by inhibiting the NLRP3 inflammasome pathway. (A) ELISA kits were used to detect the secretion levels of inflammatory cytokines IL-1β and IL-18 in the serum of mice; (B,C) WB was used to detect the protein level of cell pyroptosis markers mediated by NLRP3 inflammasome in mouse kidney tissue. Data were expressed as mean ± *SD*, *N* = 6. One-way ANOVA was used for data comparison among multiple groups and Tukey’s test was used for the *post-hoc* test. **p < 0.01, ***p < 0.001.

DN is an inflammatory disease, and pyroptosis caused by excessive inflammatory response is closely related to the onset and progression of DN [21,46]. To delineate whether TP can reduce the inflammatory response in DN renal tissue, we determined the levels of inflammatory cytokines IL-1β and IL-18 in the serum of mice. As documented in Figure 4(A), the secretion levels of IL-1β and IL-18 in the DN group were visibly higher than those in the NC group, while decreased after TP treatment. The IL-1β and IL-18 are downstream inflammatory factors regulated by the NLRP3 inflammasome pathway. To ascertain the effect of TP on the NLRP3 inflammasome pathway, we further measured the levels of related proteins by WB. The protein levels of NLRP3, ASC, pro-Caspase-1, and GSDMD-N were up-regulated in DN mice but diminished upon TP treatment (Figures 4(B,C), *p* < 0.05). In addition, DMSO unleashed no evident impact on DN mice, and there was no significant difference in each index between the DN + TP group and the DN + DAPA group (Figures 4(A–C), all *p* > 0.05). In short, TP may inhibit the inflammatory response by governing the NLRP3 inflammasome pathway, thereby reducing pyroptosis of renal tissue in DN mice.

### TP attenuated MPC5 cell damage induced by HG

**Figure 5.**
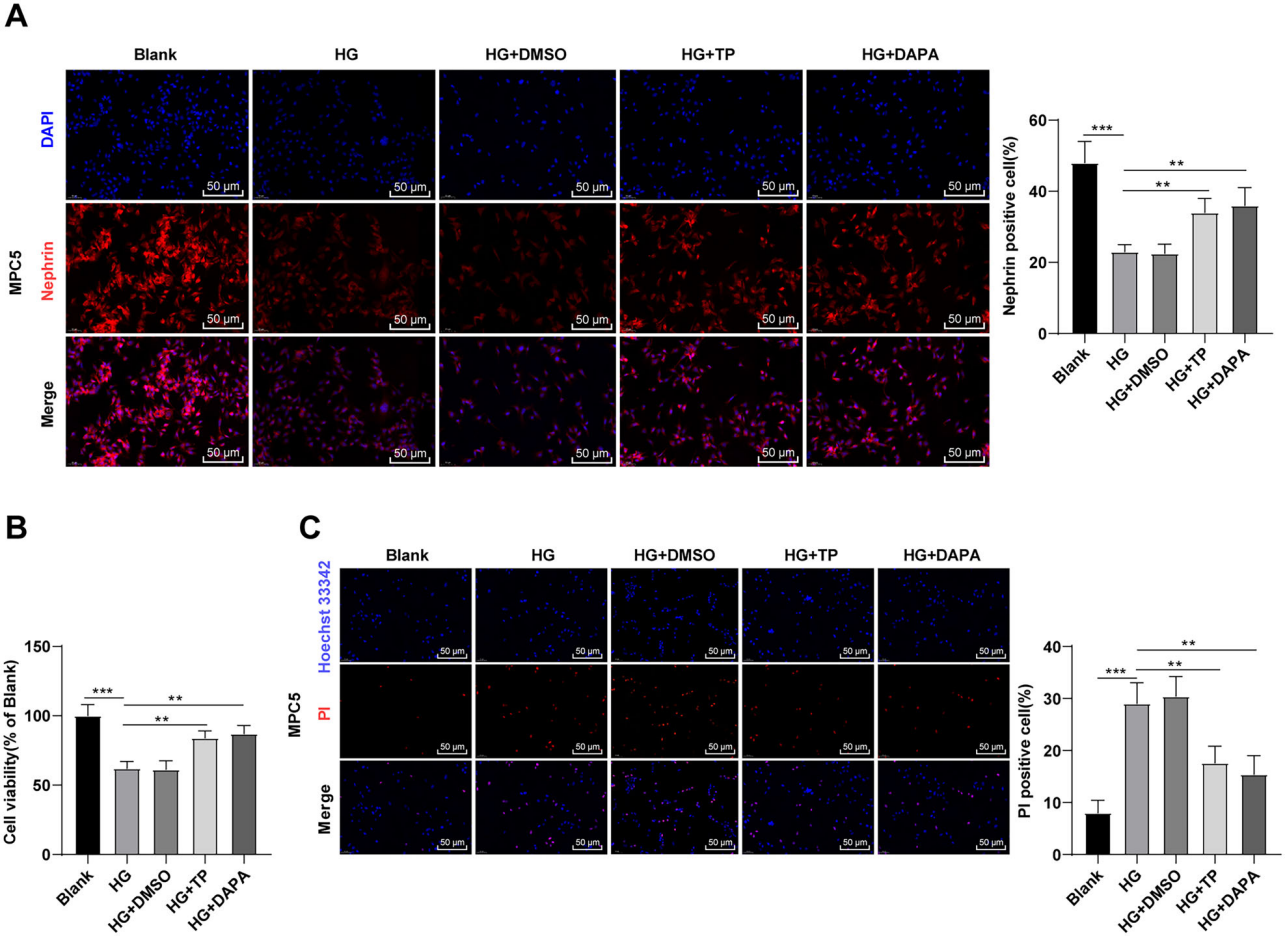
TP attenuated MPC5 cell damage induced by HG. (A) The positive expression of podocyte marker Nephrin in MPC5 cells was detected by immunofluorescent staining; (B) MPC5 cell viability was detected by MTT assay; (C) Hoechst 33342/PI double fluorescence staining was used to detect the pyroptosis level of MPC5 cells. Data were expressed as mean ± *SD*, and the cell experiments were independently repeated three times. One-way ANOVA was used for data comparison among multiple groups and Tukey’s test was used for the *post-hoc* test. **p < 0.01, ***p < 0.001.

The *in vitro* cell model of DN was established by treating MPC5 cells with HG [47], followed by intervention with TP, with DAPA as a positive control drug. Subsequently, immunofluorescent staining revealed that HG treatment reduced the level of Nephrin in MPC5 cells, while TP treatment partially restored it (Figure 5(A), all *p* < 0.01). MTT assay was adopted to testify cell proliferation and Hoechst 33342/PI double fluorescence staining was utilized to examine pyroptosis. Compared to the blank group, the cell viability of the HG group was reduced (Figure 5(B), *p* < 0.001), and the number of PI-positive cells was up-regulated (Figure 5(C), *p* < 0.001). After TP treatment, the viability of MPC5 cells was increased and PI-positive cells were decreased (Figures 5(B,C), *p* < 0.01). Meanwhile, DMSO caused no obvious effect on HG-treated MPC5 cells, and the therapeutic effect of TP was not significantly different from that of DAPA (Figures 5(A–C), all *p* > 0.05). Overall, TP can alleviate HG-induced podocyte injury.

### TP activated the Nrf2 pathway to reduce HG-induced OS in MPC5 cells

**Figure 6.**
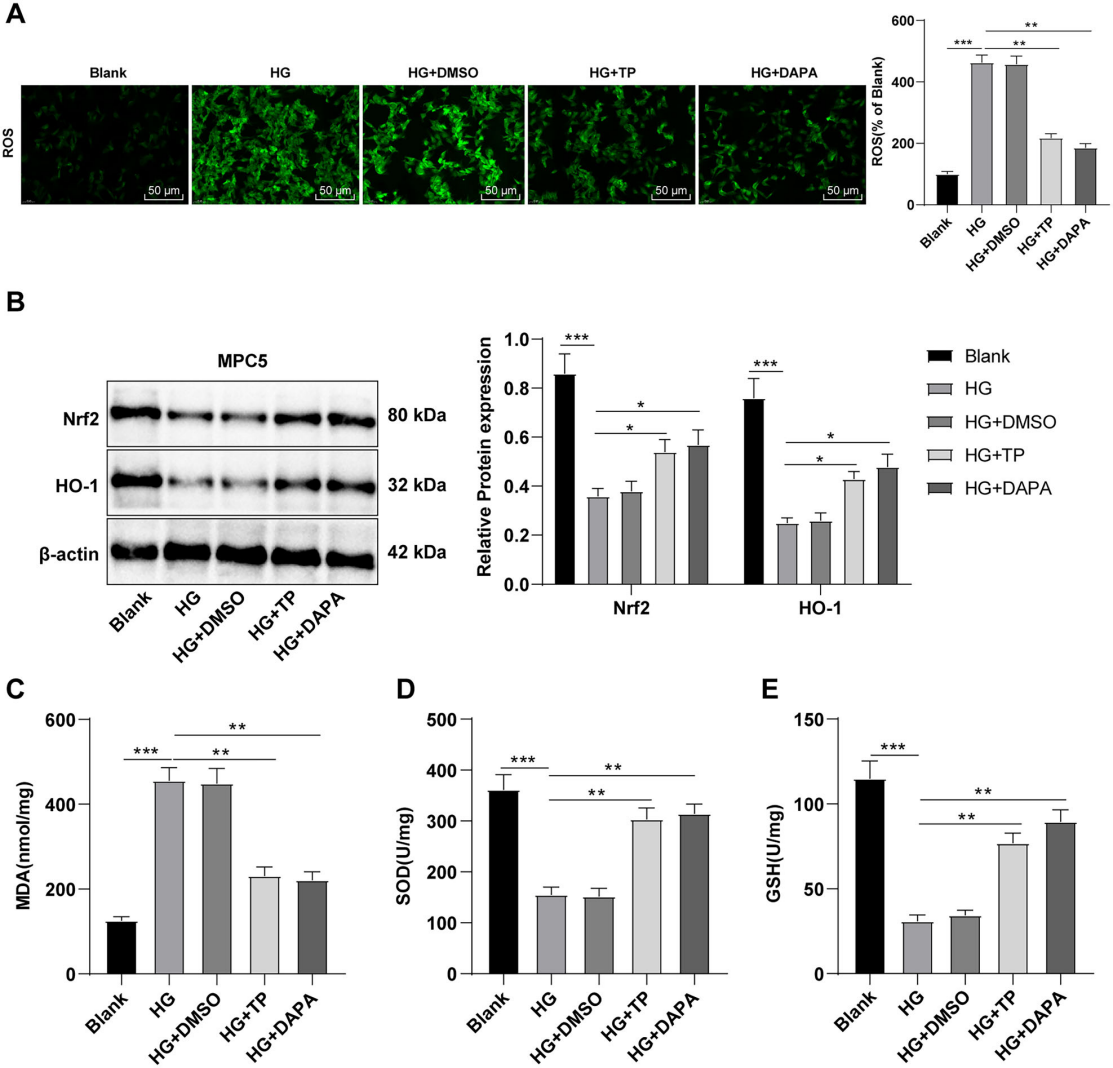
TP activated the Nrf2 pathway to reduce OS induced by HG in MPC5 cells. (A) ROS levels in MPC5 cells were detected by a DCFH-DA fluorescence probe; (B) WB was used to detect Nrf2 and HO-1 protein levels; (C–E) The MDA, SOD, and GSH levels were determined. Data were expressed as mean ± *SD*; cell experiments were independently repeated three times. One-way ANOVA was used for data comparison among multiple groups and Tukey’s test was used for the *post-hoc* test. *p < 0.05, **p < 0.01, ***p < 0.001.

Subsequently, the detection with DCFH-DA fluorescence probe revealed that HG treatment up-regulated ROS levels in MPC5 cells, while TP and DAPA both inhibited ROS levels (Figure 6(A), *p* < 0.01). WB assay revealed that Nrf2 and HO-1 levels were downregulated in the HG group but up-regulated by treatment of TP or DAPA (Figure 6(B), all *p* < 0.05). Relative to the blank group, MDA level in the HG group was enhanced, while SOD and GSH levels were reduced (Figures 6(C–E), all *p* < 0.05). However, after TP or DAPA treatment, MDA levels decreased while SOD and GSH levels increased (Figures 6(C–E), all *p* < 0.05). Likewise, DMSO resulted in no evident impact on HG-treated MPC5 cells, and there was no significant difference in the therapeutic effect between TP and DAPA. In brief, TP alleviated OS injury of MPC5 cells induced by HG by reducing ROS levels through the Nrf2/HO-1 pathway.

### TP attenuated pyroptosis of HG-induced MPC5 cells by blocking the NLRP3 inflammasome pathway

**Figure 7.**
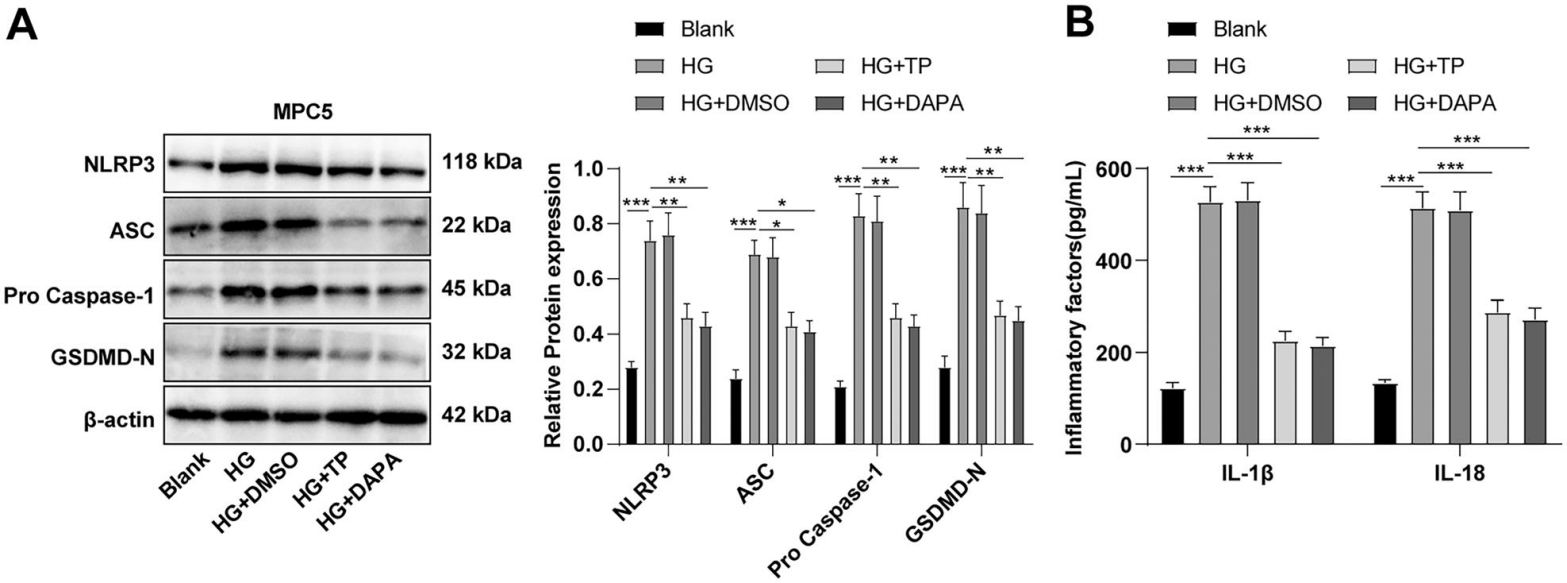
TP alleviated pyroptosis in HG-induced MPC5 cells by inhibiting the NLRP3 inflammasome pathway. (A) WB was used to detect the protein levels of pyroptosis markers in NLRP3 inflammasome in MPC5 cells; (B) The secretion levels of IL-1β and IL-18 were detected by ELISA kits. Data were expressed as mean ± *SD*, and cell experiments were independently repeated three times. One-way ANOVA was used for data comparison among multiple groups and Tukey’s test was used for the *post-hoc* test. *p < 0.05, **p < 0.01, ***p < 0.001.

The NLRP3 inflammasome-related protein levels were determined by WB, which demonstrated that NLRP3, ASC, pro-Caspase-1, and GSDMD-N levels in the HG group were higher than those in the blank group, while these protein levels in the TP + HG group were lower than those in the HG group (Figure 7(A), all *p* < 0.05). ELISA discovered that the secretion levels of IL-1β and IL-18 were up-regulated in the HG group but reduced after TP intervention (Figure 7(B)). DMSO had no significant effect on HG-treated MPC5 cells, and there was no significant difference in the therapeutic effect between TP and DAPA (Figures 7(A,B), all *p* > 0.05). These results suggest that TP can reduce pyroptosis and inflammatory damage induced by HG in MPC5 cells by suppressing the NLRP3 inflammasome pathway.

### TP protected HG-treated MPC5 cells by activating Nrf2 to scavenge ROS and inhibit the NLRP3 inflammasome pathway

**Figure 8.**
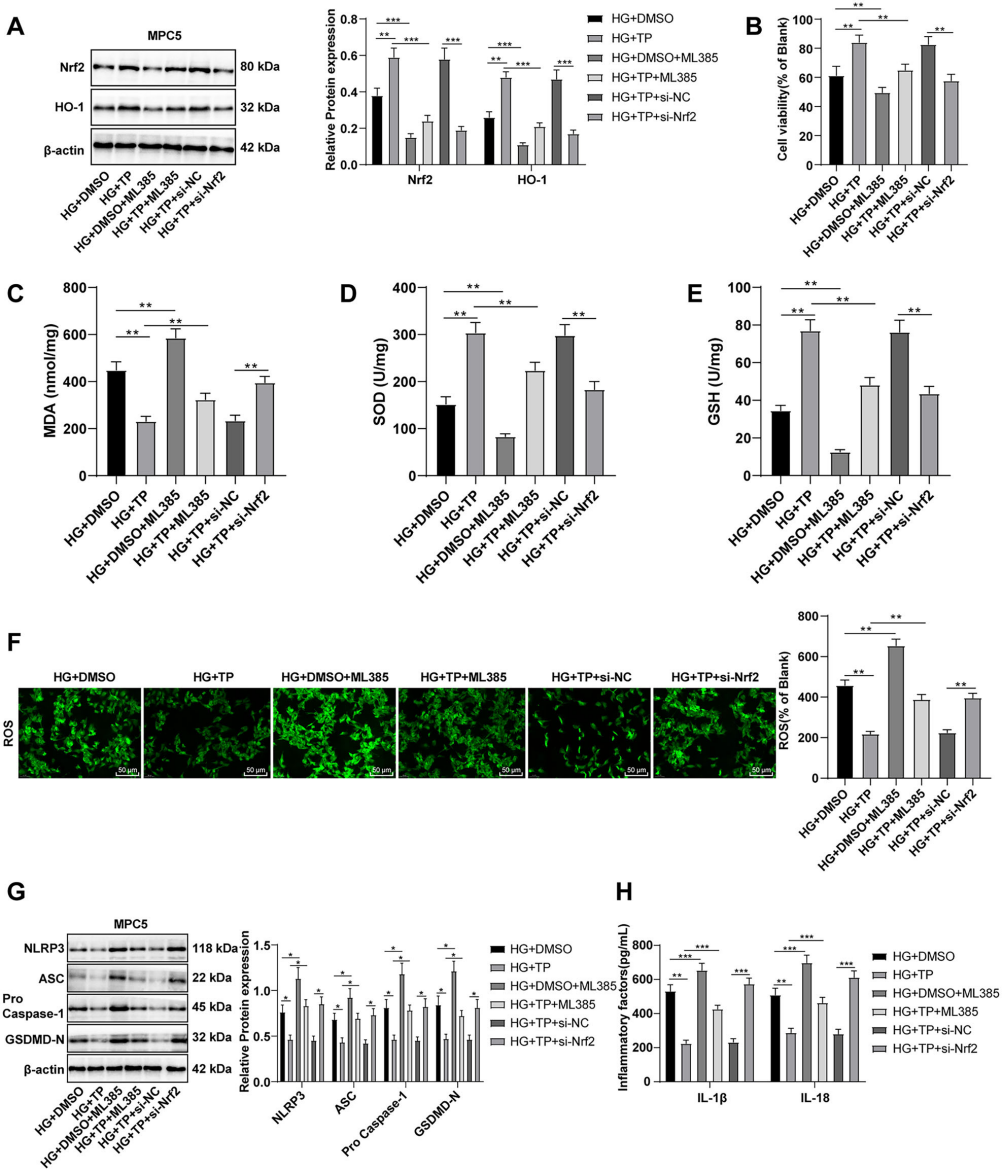
TP protected HG-treated MPC5 cells by activating Nrf2 to scavenge ROS and inhibit the NLRP3 inflammasome pathway. (A) The protein levels of Nrf2 and HO-1 in MPC5 cells were detected by WB; (B) MPC5 cell activity was detected by MTT; (C–E) The levels of MDA, SOD, and GSH in MPC5 cells were detected; (F) ROS levels in MPC5 cells were detected by a DCFH-DA fluorescence probe; (G) WB was used to detect the levels of NLRP3 inflammasome pathway-related proteins; (H) The secretion levels of inflammatory cytokines IL-1β and IL-18 were detected by ELISA; Data were expressed as mean ± *SD*, and the cell experiments were independently repeated three times. Comparison between two groups was performed by the *t*-test. *p < 0.05, **p < 0.01, ***p < 0.001.

To further verify whether Nrf2 is a target of TP, MPC5 cells were treated with the Nrf2 inhibitor ML385. WB discovered that the HG + DMSO + ML385 group had lower protein levels of Nrf2 and HO-1 than the HG + DMSO group, and the HG + TP + ML385 group also had lower levels than the HG + TP group (Figure 8(A), all *p* < 0.001), indicating that Nrf2 pathway was inhibited by ML385. After ML385 treatment, cell viability was reduced (Figure 8(B), all *p* < 0.01), MDA levels were enhanced, SOD and GSH levels were decreased (Figures 8(C–E), *p* < 0.01), and ROS levels were elevated (Figure 8(F), *p* < 0.001). These results evinced that ML385 inhibited the activation of the Nrf2 pathway induced by TP and increased ROS levels. Additionally, after ML385 treatment, the protein levels of NLRP3, ASC, pro-Caspase-1, and GSDMD-N were up-regulated (Figure 8(G), *p* < 0.001) and the levels of IL-1β and IL-18 were elevated (Figure 8(H), *p* < 0.01). MPC5 cells were transfected with si-Nrf2 and treated with a combination of HG and TP. The results unraveled that si-Nrf2 reversed the protective effect of TP on MPC5 (Figures 8(A–H), all *p* < 0.05). The above results evinced that TP could impede the NLRP3 inflammasome pathway by activating Nrf2 and scavenging ROS, thereby protecting HG-treated MPC5 cells.

## Discussion

DN, as a major complication of diabetes, is the leading cause of end-stage renal disease worldwide [48]. Recently, the functional and structural abnormalities of glomerular podocytes have been identified as one of the earliest events in the development of diabetic glomerular injury [49–51]. It is worth noting that TP could considerably reduce proteinuria and podocyte injury in DN animal models [52]. Our finding illustrated that TP inhibited OS and cell pyroptosis through the Nrf2/ROS/NLRP3 regulatory axis to protect against podocyte injury in DN.

Indeed, the protective effect of TP on DN has been elucidated in many reports [53–55]. Yet, the behind mechanism has not been fully elucidated so far. By treating mice with HFD combined with STZ injection and inducing MPC5 cells with HG, the DN mouse model and cell model were established, followed by treatment with TP. DN is a serious kidney disease characterized by kidney injury and tissue fibrosis [56]. In our current study, we reproduced the typical pathological changes in DN mouse kidneys, and TP treatment alleviated renal dysfunction and histopathological damage in DN mice, consistent with the reported protection of TP against renal histopathological injury in diabetic rats [25,57,58]. Several reports have shown the essential role of podocytes in the pathological mechanism of DN [43,59]. Specifically, podocyte loss and injury frequently occur in early DN patients, which may lead to severe proteinuria and renal damage [60,61]. Expectedly, we observed evident damage of renal podocytes in DN mice, while all indexes were recovered after TP treatment, with enhanced Nephrin and Podocin levels, consistent with the reported protection of TP on glomerular podocytes in DN rats [26,52].

Accumulated ROS may interact with fatty acids (polyunsaturated), leading to the formation of lipid peroxidation in renal tissues, which may ultimately lead to damage and toxicity [62]. On the other hand, OS is widely acknowledged as a major factor in DM-related complications, including DN [63,64], while TP is widely known for its good antioxidant effects [65,66]. The current study found that ROS levels and MDA levels in DN mice and HG-induced MPC5 cells were diminished after TP treatment, while SOD and GSH levels were increased, consistent with the prior finding that TP can visibly reduce renal inflammation and OS level in DN mice [67]. Overall, TP alleviated the OS injury of kidney tissue in DN mice and HG-induced MPC5 cells. On a separate note, the Nrf2/HO-1 pathway is crucial in anti-oxidant stress [44,45]. Subsequent experimentation in our study revealed that the levels of Nrf2 and HO-1 in DN mice and HG-induced MPC5 cells were decreased, whereas they were up-regulated after TP treatment. TP is capable of reducing the production of ROS and M1-type polarization by activating the Nrf2/HO-1 pathway in inflammatory bowel diseases [68]. Meanwhile, TP alleviates myocardial ischemia/reperfusion injuries in rats by activating the Nrf2/HO-1 pathway [69]. For the first time, our results reveal that TP may protect against DN by activating the Nrf2/HO-1 pathway to reduce ROS levels and OS injury.

Pyroptosis caused by an excessive inflammatory response is closely associated with DN [21,46]. Increased levels of inflammatory cytokines IL-1β and IL-18 are found in DN podocytes [70]. As expected, TP intervention diminished the secretion levels of IL-1β and IL-18 in DN mice and HG-induced MPC5 cells. TP can improve DN by regulating Th1/Th2 cell balance and reducing macrophage infiltration and levels of related-inflammatory factors in the kidney [71]. NLRP3 inflammasome may be involved in DN through activation of pyroptosis, and IL-1β and IL-18 are downstream inflammatory cytokines regulated by the NLRP3 inflammasome pathway [21,46,72]. Besides, the accumulation of ROS activates the NLRP3 pathway [73]. It is interesting to note that TP treatment partially reduced the protein levels of NLRP3 inflammasome-mediated pyroptotic markers in DN mice and HG-induced MPC5 cells. Likewise, TP induces GSDME-mediated pyroptosis of head and neck tumor cells by inhibiting mitochondrial hexokinase-ΙΙ [74]. Moreover, TP could prevent IgAN progression [75] and improve myocardial fibrosis by down-regulating NLRP3 inflammasomes [76]. Overall, this study strengthens the idea that TP may inhibit the inflammatory response and reduce the pyroptosis of renal podocytes in DN through the NLRP3 inflammasome pathway.

Nrf2 is a key transcription factor for cell regulation of OS, which can activate the transcription and expression of downstream anti-OS-related enzymes, such as HO-1 and SOD to eliminate the abnormal accumulation of ROS in cells, thereby alleviating OS injury [77,78]. ROS is crucial in the stimulation of NLRP3 inflammasome, and suppression of ROS levels in cells can inhibit the stimulation of NLRP3 inflammasome [21,23]. It can therefore be assumed that TP protects renal podocytes by activating the Nrf2 pathway to reduce ROS levels and inhibit the NLRP3 inflammasome pathway. Nrf2 inhibitor ML385 can eliminate COQ10-mediated renal protection [38]. Not surprisingly, our study illustrated that ML385 inhibited Nrf2 and raised ROS levels and si-Nrf2 reversed the protective effect of TP on MPC5 cells. This observation may support the hypothesis that TP alleviated inflammatory damage and pyroptosis of podocytes in DN by regulating the Nrf2/ROS/NLRP3 axis.

Previous studies have confirmed that DAPA can attenuate STZ-induced DN [79,80], so we chose DAPA as a positive control drug for TP. In our studies, the therapeutic effect of TP was not significantly different from that of DAPA, which indicated the potential of TP as a candidate drug for the treatment of DN. DAPA is an SGLT2 inhibitor with a single site of action. In contrast to DAPA, TP may be a multi-target therapeutic agent for DN. For instance, TP can inhibit the PDK1/Akt/mTOR pathway to restrain glomerular mesangial cell proliferation in DN [30]. TP impedes extracellular matrix accumulation in experimental DN by targeting the microRNA-137/Notch1 pathway [57]. TP alleviates podocyte epithelial-mesenchymal transition in DN *via* the kindlin-2 and EMT-related TGF-β/Smad pathway [26]. The aforementioned evidence has further evinced the superiority of TP as a therapeutic candidate for DN.

## Conclusion

To conclude, through animal and cell experiments, this study highlighted for the first time that TP protected podocytes from OS and pyroptosis in DN by activating the Nrf2 pathway and inhibiting the NLRP3 inflammasome pathway, which further clarified the mechanism of TP in reducing DN and provided references and therapeutic targets for new therapeutic drugs for DN. In the future, we will conduct more animal experiments and explore the direct regulation of TP on Nrf2 and NLRP3 in cell experiments, and further explore whether TP can protect against DN through epigenetic regulation, hoping to further clarify the protective mechanism of TP in DN in clinical application

## Data Availability

The data that support the findings of this study are available from the corresponding author upon reasonable request.
